# A Mass Balance Approach for Thermogravimetric Analysis in Pozzolanic Reactivity R^3^ Test and Effect of Drying Methods

**DOI:** 10.3390/ma14195859

**Published:** 2021-10-07

**Authors:** Kira Weise, Neven Ukrainczyk, Eduardus Koenders

**Affiliations:** Institute of Construction and Building Materials, Technical University of Darmstadt, 64287 Darmstadt, Germany; koenders@wib.tu-darmstadt.de

**Keywords:** pozzolanic reactivity R^3^ test, supplementary cementitious materials (SCMs), pozzolan, calcined clay, metakaolin, silica fume, thermogravimetric analysis (TGA), isopropanol, acetone, reaction calorimetry

## Abstract

The reactivity of supplementary cementitious materials (SCMs) is a key issue in the sustainability of cement-based materials. In this study, the effect of drying with isopropanol and acetone as well as the interpretation of thermogravimetric data on the results of an R^3^ test for evaluation of the SCM pozzolanic reaction were investigated. R^3^ samples consisting of calcium hydroxide, potassium hydroxide, potassium sulphate, water, and SCM were prepared. Besides silica fume, three different types of calcined clays were investigated as SCMs. These were a relatively pure metakaolin, a quartz-rich metakaolin, and a mixed calcined clay, where the amount of other types of clays was two times higher than the kaolinite content. Thermogravimetric analysis (TGA) was carried out on seven-day-old samples dried with isopropanol and acetone to stop the reaction processes. Additional calorimetric measurement of the R^3^ samples was carried out for evaluation of the reaction kinetics. Results show that drying with isopropanol is more suitable for analysis of R^3^ samples compared to acetone. The use of acetone results in increased carbonation and TGA mass losses until 40 (isothermal drying for 30 min) and 105 °C (ramp heating), indicating that parts of the acetone remain in the sample, causing problems in the interpretation of TGA data. A mass balance approach was proposed to calculate calcium hydroxide consumption from TGA data, while also considering the amount of carbonates in the sample and TGA data corrections of original SCMs. With this approach, an improvement of the linear correlation of TGA results and heat release from calorimetric measurement was achieved.

## 1. Introduction

Concrete with cement as binder is still one of the most used construction materials worldwide [1], which is also reflected by enormous global cement production, which was estimated at around four billion tons in 2018 [2]. The massive production of ordinary Portland cement (OPC) is responsible for emitting enormous amounts of androgenic CO_2_ caused by both fossil fuel burning and decarbonation of limestone [3]. Lowering these impacts can generally be achieved by lowering the amounts of cement clinker in concrete and/or by replacing it with more environmentally friendly alternatives [4]. Common supplementary cementitious materials (SCMs), such as fly ash, silica fume, or ground granulated blast-furnace slag, are mainly employed for this purpose. However, the availability of fly ash as well as ground granulated blast-furnace slag is becoming rare due to a worldwide reduction of the number of coal-fired electricity plants and shortages on the world market, respectively [5]. From this, it has become clear that the search for alternative SCMs for partial replacement of OPC is vital. Possible alternative SCM materials for their use in cementitious systems strongly depend on their potential reactivity, which drives the need for descriptive reactivity tests. For this reason, different testing procedures are used for measuring the reactivity of SCMs, as there is still no universally valid testing procedure available. To meet the general interest for a rapid, relevant, and reliable test, AVET et al. first introduced the R^3^ test for evaluation of the pozzolanic reactivity of calcined kaolinitic clays in 2016 [6]. They developed a test environment for the pore solution of cementitious systems by using calcium hydroxide, potassium hydroxide, potassium sulphate, and water [6]. Bound water was determined using oven thermal treatment and heat flow measurements were carried out by using isothermal calorimetry on R^3^ samples [6]. This approach enabled evaluation of the pozzolanic reaction of SCMs independently of the specific cement properties. In the following years, the R^3^ test was developed further and extended with additional testing procedures like portlandite consumption measured with thermogravimetric analysis (TGA) and chemical shrinkage [7]. The R^3^ test was employed for evaluating the reaction behavior of many different kinds of SCM, such as, besides calcined clays, ground granulated blast-furnace slag, silica fume, and fly ash [7,8,9,10,11]. 

For some of these reactivity test methods, e.g., TGA, drying of the samples is needed to stop the reaction processes prior to analysis. In a round robin test carried out by Snel-lings et al. different stoppage methods were tested on cement paste samples [12]. The solvent exchange method was found to perform best regarding the composition and preservation of pores in cementitious samples compared to oven, vacuum, and freeze drying [12,13]. Zhang et al. compared different solvents for the drying of cement paste samples prior to the thermogravimetric analysis [14]. In their study, the order of mass loss in the temperature range of 600 to 1000 °C that refers to carbonates was the following: Acetone > ethanol > tetrahydrofuran > isopropanol [14]. In a similar study, Röser chose acetone treatment, against drying with isopropanol, freeze drying, and microwave drying, as the best possible method for hydration stoppage of cement paste samples for thermogravimetric analysis [15].

For the reaction stoppage of R^3^ samples, some research groups [7,11,16,17] followed the drying procedure with isopropanol suggested by Snellings et al. for cement paste samples [18]. Blotevogel et al. used a combination of isopropanol and acetone treatment to stop the reaction processes in R^3^ samples [8]. Additionally, in contrast, Suraneni and Weiss did not use any drying procedure in their study as they calculated the amount of Ca(OH)_2_ only, as any hydration stoppage would complicate the interpretation of the test results [9]. 

As various drying methods for R^3^ samples have been employed in the literature, this study aimed to compare the effect of two different drying procedures with acetone and isopropanol on the thermogravimetric results of the R^3^ test regarding mainly calcium hydroxide consumption. The samples were prepared, dried and analyzed in a well-defined testing program that followed strict time tables, which is especially relevant for drying and thermogravimetric testing of the samples. Calorimetric measurement of the R^3^ samples was used for evaluation of the thermogravimetric test results.

The work starts with a detailed explanation of the procedure needed for interpretation of thermogravimetric data from the R^3^ test, following a precise description of the mass balance approach. Calcium hydroxide consumption was calculated according to a procedure that both takes into account the amount of carbonates in the sample and TGA data corrections of original SCMs. 

## 2. Materials and Methods

### 2.1. Materials

In the experimental program, samples for the R^3^ tests were prepared containing R^3^ emulsion and different SCMs. The SCMs employed in this study were silica fume (SF) and three different types of calcined clay, which were a relatively pure metakaolin (MK1), another with 46 wt.% metakaolin and around 40 wt.% quartz impurities (MK2), and finally, a mixed calcined clay (MC) with 25 wt.% kaolinit in the raw material. The chemical composition of the used SCMs is shown in Table 1. 

The used silica fume (SF) contains 97 wt.% of SiO_2_ (Table 1). Rietveld refinement of X-ray diffraction using Topas version 5 software from Bruker (Billerica, MA, USA) spiked with 10% corundum resulted in 91 wt.% amorphous material, with a specific density of around 2.2 g/cm^3^ and a specific surface of 150,000–300,000 cm^2^/g stated in the Elkem Data Sheet [19]. The mean particle size is around 0.15 µm.

**Figure 1:**
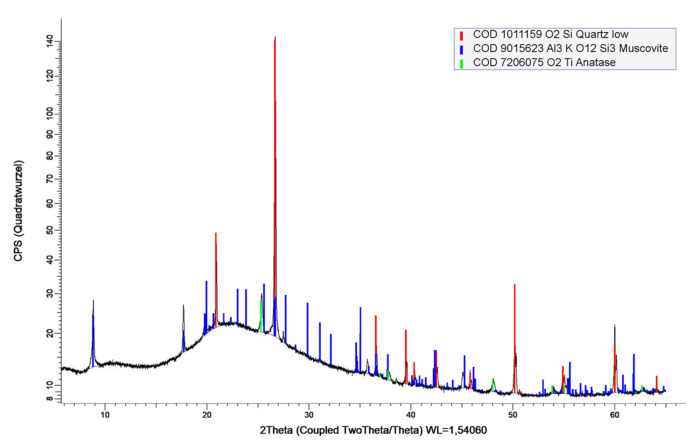
Powder X-ray diffractogram and qualitative analysis of metakaolin (MK1).

The relatively pure industrial type of metakaolin MK1 is produced by grinding calcined kaolinite-rich clay originating from secondary geological deposits (not known), with a specific density of 2.6 g/cm^3^ and a specific surface of 200,000 cm^2^/g (BET). MK1 is a commercial product produced by industrial-scale calcination and grinding. It was calcined in an industrial-scale Herreshoff furnace (multiple-hearth), where, in each hearth, the temperature and time of calcination were precisely controlled (assumed to be <~750 °C) to ensure high reactivity. The corresponding qualitative X-ray diffraction result is shown in Figure 1, while the Rietveld refinement analysis resulted in an amorphous content of 83 wt.% (~metakaolin), 10 wt.% quartz, 5 wt.% muscovite, and 2 wt.% anatase. 

**Figure 2:**
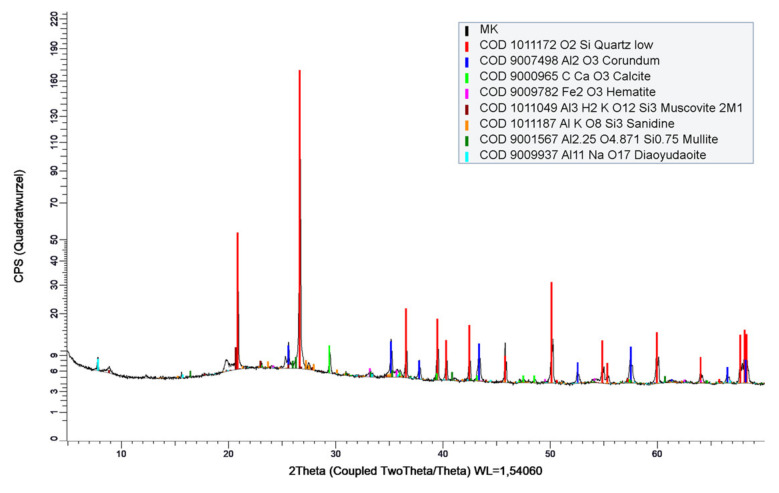
Powder X-ray diffractogram and qualitative analysis of metakaolin (MK2).

The second metakaolin MK2 is less pure compared to MK1 and contains impurities mainly of quartz. The qualitative X-ray diffraction diagram is shown in Figure 2, resulting in 46 wt.% amorphous material (~metakaolin), 40 wt.% quartz, 10 wt.% muscovite, and 2 wt.% calcite. Moreover, MK2 has a specific density of 2.5 g/cm^3^ and a specific surface of 160,000 cm^2^/g (BET).

The raw clay used for the mixed calcined clay MC used was mined in the region of Unterstürmig in northern Bavaria (south part of Germany) and is a 180 million-year-old clay from the age of Lias. It was calcined industrially in a rotary kiln at around 650 °C and milled afterwards (more details can be found in [20]). The raw clay consists mainly of 25 wt.% kaolinit, 30 wt.% mica, 11 wt.% illite, 6 wt.% chlorite, and 18 wt.% quartz [21], and was stored as grey-brown powder in small size bags. According to Liapor GmbH & Co. KG (Hallerndorf-Pautzfeld, Germany), the largest grain diameter of the mixed calcined clay is ≤100 µm and the amount of particles smaller than 32 µm is over 90 wt.%. The specific density is 2.63 g/cm^3^, bulk density is 1.00 g/cm^3^, and the specific surface area according to BET is 55,000 cm^2^/g. X-ray diffraction indicates a persistence of illite and mica (muscovite) peaks, showing that kaolinite is transformed to an amorphous material during calcination, but 2:1 of the clay stays semi-crystalline. 

### 2.2. Sample Preparation

Samples for the R^3^ tests were mixed with an alkaline emulsion (R^3^ emulsion) proposed by Li et al. and Avet et al. that mimics the alkaline pore solution of cement paste [6,7]. The R^3^ emulsion contains powdered calcium hydroxide (≥96%), potassium hydroxide in flakes (≥85%), powdered potassium sulphate (≥99%), and deionized water. The composition is shown in Table 2. Firstly, potassium hydroxide flakes (KOH) were dissolved in 7 g of deionized water for 15 min using a magnetic stirrer. In a second step, dissolved potassium hydroxide was homogenized with the remaining constituents (calcium hydroxide, potassium sulphate, and the remaining deionized water) using an electric mixer for 15 min. 

Subsequently, 100 g of alkaline R^3^ emulsion were mixed with 12.59 g of powder SCM for five minutes using an electric stirrer. The ratio, i.e., 12.59/100, was taken from the investigations of Avet et al. [6]. Additionally, a reference mix was prepared containing R^3^ emulsion only (without SCM). The mixed lime for TGA measurements was poured in 10 ml sealed polypropylene containers for each SCM and stored in an oven at 40 °C for seven days. After that, the reaction process was stopped by drying the samples with two different solvents (isopropanol and acetone). For each SCM, three samples were dried with isopropanol and three with acetone. 

The drying procedure with isopropanol was carried out according to Snellings et al. [12,18]. R^3^ samples of 3 g ± 0.05 g were crushed to pieces smaller than 1 mm and immersed in 100 mL of isopropanol for 15 min while stirring the suspension manually with the help of a glass stick. In the next step, the suspension was poured gently on a Büchner filter, letting the isopropanol percolate. The residue was rinsed with 20 mL of isopropanol and twice with 20 mL of diethyl ether. The residue was placed on a watch glass and dried for 8 min ± 30 s in an oven at 40 °C. 

For the drying procedure with acetone, 3 g ± 0.05 g of the R^3^ sample was gently hand-milled and washed three times with 5 mL of acetone (>99.5%) while continuing milling according to Röser and Weise [15,22,23]. For the reference sample (R^3^ emulsion without SCM), seven aliquots of 5 mL of acetone were needed to dry the samples. 

Prior to analysis, the samples were stored in a desiccator over silica gel and soda lime according to Snellings et al. [18]. Isopropanol samples were gently hand-milled just before thermogravimetric testing (<5 min). 

The samples dried with isopropanol were indicated as “ISO” and with acetone “ACT”. They were numbered according to the order of sample preparation and TGA measurement. The samples ISO_1 and ACT_1 were tested on the same day as the reaction stoppage. Thermogravimetric analysis of both samples ISO_2, ACT_2, ISO_3, and ACT_3 was conducted the next day, respectively, which was in line with the recommendations of Snellings et al. [18]. The order of TGA measurement was the following: ISO_1, ACT_1, ISO_2, ACT_2, ISO_3, and ACT_3. The used original materials (SCMs) were measured with thermogravimetric analysis separately.

For the calorimeter tests, lime samples were mixed as described above and directly placed in the calorimeter chamber, which was preconditioned at 40 °C. The heat release was measured for a duration of 7 days.

### 2.3. Measurement Methods

For analyzing the reaction processes of the R^3^ samples, the samples were tested both by TGA and by a calorimeter. 

For thermogravimetric analysis (TGA), “STA 449 F5 Jupiter” from NETZSCH (Selb, Germany) was employed. The crucibles consisted of alumina and were filled with 40–50 mg of powder material. To avoid oxidation, nitrogen was used as an inert gas. The samples were first heated up to 40 °C and kept constant at this temperature for 30 min. Subsequently, each sample was heated up to 1000 °C at a constant heating rate of 20 °C per minute.

For the calorimetric measurements, “MC CAL” from C3 Prozess- und Analysentechnik GmbH (Haar, Germany) was employed. For this, 20 g of R^3^ sample were prepared at room temperature and placed in the calorimeter ampoule, which was preconditioned at 40 °C. Measurements of the heat release of the R^3^ samples were carried out for seven days. The reference sample was used for the calculation of the specific heat capacity.

### 2.4. A Mass Balance Approach for TGA in Pozzolanic Reactivity R^3^ Test

For the interpretation of thermogravimetric data, the sample mass inside the sealed container was assumed to remain constant during the reaction processes. Pozzolanic reactions of SCM turn parts of the calcium hydroxide and water into hydration phases. As a result, reaction products are formed that contain a certain amount of chemically bounded water. As calcium carbonate was detected in the R^3^ samples, mainly resulting from the drying procedure of the samples after seven days, these were considered for the interpretation of the test results as well. Determination of the actual amount of carbonates in the sample is needed to exactly calculate the consumption of calcium hydroxide by SCMs [24]. Based on these simplified schematic considerations, a mass balance approach from the thermogravimetric data of the lime samples containing SCM, water, calcium hydroxide, potassium hydroxide, and potassium sulphate is subsequently proposed. 

In the first step, all thermogravimetric data are corrected to the measured mass at 40 °C (*m40*) according to Equation (1), so that the 100 wt.% sample is related to a temperature of 40 °C:

(1) $$x^{*}=\frac{x}{m40}\;\cdot \;100\%$$

where:

- x* is the corrected TGA value in wt.%;
- *x* is any TGA data (mass or mass loss) in wt.%;
- *m40* is the mass at 40 °C taken from TGA in wt.%.

This approach follows the assumption that mass loss from room temperature up to 40 °C is caused by remaining solvents and consequently mainly referring to the drying procedure [15]. 

In the proposed mass balance approach, the measured mass at 1000 °C (*m1000*) is used to standardize the results to the reference value of g/100 g SCM. The remaining mass at 1000 °C (*m1000*) consists of the following anhydrous components (*Anhydrous*): the anhydrous parts of unreacted SCM, anhydrous parts of reaction products, CaO from Ca(OH)_2_, K_2_SO_4_, and K from KOH. With the information of the initial masses from the testing program and the stoichiometric considerations, it is possible to calculate the amount of SCM related to *m1000* (*SCM_Anhydrous*):

(2) $$\mathrm{Anhydrous}=\frac{\mathrm{mix\_SCM}}{\mathrm{mix\_solid}}\;\cdot {\;m1000\_\mathrm{SCM}}^{*}+\frac{\mathrm{mix}\_\mathrm{Ca}\left(\mathrm{OH}\right)2}{\mathrm{mix}\_\mathrm{solid}}\;\cdot \;100\%\;\cdot \;\frac{56}{74}\;+\frac{\mathrm{mix}\_K2\mathrm{SO}4}{\mathrm{mix}\_\mathrm{solid}}\cdot \;100\%+\frac{\mathrm{mix}\_\mathrm{KOH}}{\mathrm{mix}\_\mathrm{solid}}\cdot \;100\%\;\cdot \;\frac{39}{56}$$

(3) $$\mathrm{SCM}\_\mathrm{Anhydrous}=\frac{\mathrm{mix}\_\mathrm{SCM}}{\mathrm{mix}\_\mathrm{solid}}\;\cdot \;100\%\;\cdot \;\frac{100\%}{\mathrm{Anhydrous}\;}$$

where:

- *Anhydrous* is the amount of anhydrous components in g/100 g *mix_solid* calculated according to Equation (2);
- *mix_solid* is the mass of solid components in the mix design of the testing program in g;
- *mix_y* is the mass of component y (SCM, Ca(OH)_2_, K_2_SO_4_, or KOH) in the mix design of testing program in g;
- m1000_SCM* is the corrected mass at 1000 °C taken from TGA of pure SCM in wt.%;
- *SCM_Anhydrous* is the amount of SCM in g/100 g *Anhydrous* calculated according to Equation (3).

The numbers in Equation (2) as well as in Equations (4), (5) and (7) result from the molar masses of CaO (56 g/mol), Ca(OH)_2_ (74 g/mol), K (39 g/mol), KOH (56 g/mol), H_2_O (18 g/mol), CaCO_3_ (100 g/mol), and CO_2_ (44 g/mol), respectively. 

In the next step, the corrected thermogravimetric data according to Equation (1) are analyzed to determine the amount of Ca(OH)_2_, CaCO_3_, and *w*, which summarizes the amount of physically and chemically bounded water (without water in calcium hydroxide) as well as the remaining organic solvents and free water in the sample. The results are standardized to the unit g/100 g SCM by using Equation (3). The mass loss caused by the dehydroxylation of calcium hydroxide in a temperature range of around 400 to 500 °C (in line with the literature [7,23,25,26]) was determined with the tangential method according to Lothenbach et al. [27]. The amount of CaCO_3_ was calculated according to [28,29] with the mass loss between 600 and 750 °C (in line with the literature [23,30,31,32,33,34]) using the stepwise method:

(4) $$\mathrm{Ca}{\left(\mathrm{OH}\right)}_{2}=\frac{\mathrm{ML}\_\mathrm{Ca}\left(\mathrm{OH}\right)2^{*}}{{m1000}^{*}}\;\cdot \;100\;\cdot \;\frac{74}{18}\;\cdot \;\frac{100}{\mathrm{SCM}\_\mathrm{Anhydrous}}$$

(5) $${\mathrm{CaCO}}_{3}=\frac{{\mathrm{ML}\_\mathrm{CaCO}3}^{*}}{{m1000}^{*}}\;\cdot \;100\;\cdot \;\frac{100}{44}\;\cdot \;\frac{100}{\mathrm{SCM}\_\mathrm{Anhydrous}}$$

(6) $$w=\frac{{m40}^{*}{\;-\;m600}^{*}{\;-\;\mathrm{ML}\_\mathrm{Ca}(\mathrm{OH})2}^{*}}{{m1000}^{*}}\;\cdot \;100\;\cdot \;\frac{100}{\mathrm{SCM}\_\mathrm{Anhydrous}}$$

where:

- *Ca(OH)_2_* is the amount of calcium hydroxide in the sample in g/100 g SCM;
- ML_Ca(OH)2* is the corrected mass loss between approximately 400 and 500 °C determined with the tangential method from TGA in wt.%;
- m1000* is the corrected mass at 1000 °C taken from TGA in wt.%;
- *SCM_Anhydrous* is the amount of SCM in g/100 g *Anhydrous* calculated according to Equation (3);
- *CaCO_3_* is the amount of calcium carbonate in the sample in g/100 g SCM;
- ML_CaCO3* is the corrected mass loss between 600 and 750 °C determined with the stepwise method from TGA in wt.%;
- *w* is the amount of physically and chemically bounded water (without water in calcium hydroxide) as well as the remaining organic solvents and free water in the sample in g/100 g SCM;
- m40* is the corrected mass at 40 °C taken from TGA in wt.%;
- m600* is the corrected mass at 600 °C taken from TGA in wt.%.

Pozzolanic reactivity is often described by the consumption of calcium hydroxide (*Consumption CH*). With the thermogravimetric data of an R^3^ test, it is possible to calculate this value. Consumption of Ca(OH)_2_ represents the difference between the initial Ca(OH)_2_ in a mix design and the amount of Ca(OH)_2_ in a sample at the testing time, as defined in [7,9]. Additionally, the proposed mass balance approach of this study takes into account those parts of Ca(OH)_2_ that are bound in CaCO_3_ during drying or even during heating of the thermogravimetric measurement as stated by Taylor and Turner [35]. This basic approach was already suggested before by Kim and Olek for the interpretation of thermogravimetric data of cement paste samples [24]. To calculate the amount of bound Ca(OH)_2_ in a carbonation process, the amount of CaCO_3_ in the sample according to Equation (5) needs to be corrected with the value of CaCO_3_ in the original SCM. Moreover, the possible amount of Ca(OH)_2_ in SCM reduces the consumption of Ca(OH)_2_ by SCM and needs to be taken into account according to Equation (7):

(7) $$\mathrm{Consumption}\;\mathrm{CH}=\frac{\mathrm{mix}\_\mathrm{Ca}\left(\mathrm{OH}\right)2}{\mathrm{mix}\_\mathrm{SCM}}\;\cdot \;100\;-\;\mathrm{Ca}{\left(\mathrm{OH}\right)}_{2}-{\;(\mathrm{CaCO}}_{3}-\;{\mathrm{ML}\_\mathrm{CaCO}3\_\mathrm{SCM}}^{*}\;\cdot \;\frac{100}{44})\cdot \;\frac{74}{100}-{\;\mathrm{ML}\_\mathrm{Ca}(\mathrm{OH})2\_\mathrm{SCM}}^{*}\;\cdot \;\frac{74}{18}\;\;$$

where:

- *Consumption CH* is the consumption of calcium hydroxide by the pozzolanic reaction of the SCM in g/100 g SCM;
- *mix_y* is the mass of component *y* (SCM or Ca(OH)_2_) in the mix design of the testing program in g;
- *Ca(OH)_2_* is the amount of calcium hydroxide in the sample in g/100 g SCM;
- *CaCO_3_* is the amount of calcium carbonate in the sample in g/100 g SCM;
- ML_CaCO3_SCM* is the corrected mass loss between 600 and 750 °C determined with the stepwise method from TGA of original SCM in wt.%;
- ML_Ca(OH)2_SCM* is the corrected mass loss between approximately 400 and 500 °C determined with the tangential method from TGA of original SCM in wt.%.

For reasons of comparability, *Consumption CH* was also calculated without considering the carbonation processes and TGA data of the original SCM according to Equation (8):

(8) $$\mathrm{Consumption}\;\mathrm{CH}\;(\mathrm{Common})\;=\;\frac{\mathrm{mix}\_\mathrm{Ca}\left(\mathrm{OH}\right)2}{\mathrm{mix}\_\mathrm{SCM}}\;\cdot \;100\;-\;\mathrm{Ca}{\left(\mathrm{OH}\right)}_{2}$$

where:

- *Consumption CH (Common)* is the consumption of calcium hydroxide by the pozzolanic reaction of the SCM in g/100 g SCM calculated without considering the carbonation and TGA data of pure SCM;
- *mix_y* is the mass of component y (SCM or Ca(OH)_2_) in the mix design of the testing program in g;
- *Ca(OH)_2_* is the amount of calcium hydroxide in the sample in g/100 g SCM.

## 3. Results and Discussion

**Figure 3:**
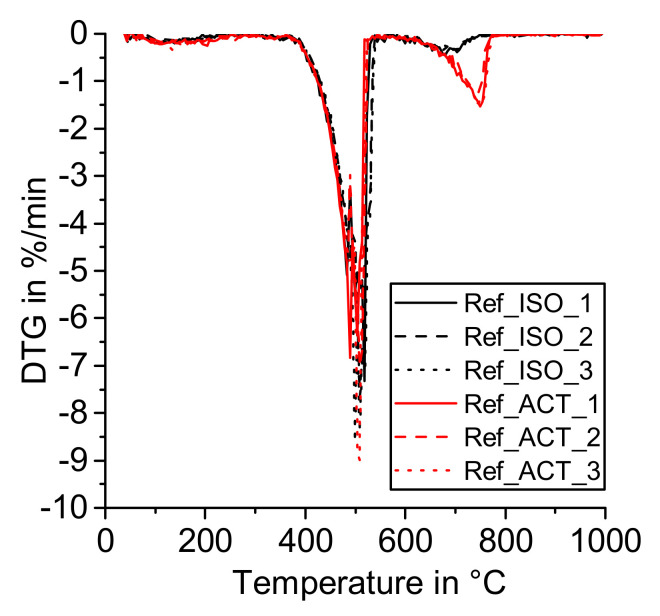
DTG—Reference (R^3^ emulsion without SCM) dried with isopropanol (ISO) and acetone (ACT).

The derivative of a TG curve (DTG) of the reference sample indicates that drying with acetone results in an increased amount of carbonates (mass loss from 600 to 750 °C, Figure 3) compared to the samples dried with isopropanol according to the procedures described in Section 2.2. 

Evaluation of the reference sample results in a calculated amount of calcium carbonate (*CaCO_3_*) of 9.85 wt.% with acetone drying and 3.39 wt.% when isopropanol was used to stop the reaction. The amount of calcium hydroxide (*Ca(OH)_2_*) in the samples was 83.48 wt.% for acetone drying and 91.63 wt.% with isopropanol. The larger amount of CaCO_3_ and the decrease in Ca(OH)_2_ with the use of acetone for drying was already reported earlier by Taylor and Turner [35]. They observed an aldol condensation reaction of acetone in an alkaline environment at room temperature. The sorbed and newly formed organic substances are less volatile than acetone and yield carbonate ions upon heating during a thermogravimetric measurement (200 to 600 °C) [27,35,36]. Kim and Olek pointed out that drying cement paste samples before grinding, as it was done in the isopropanol treatment in this study, results in less carbonation compared to drying samples after grinding, like the acetone treatment described in this paper [24]. The sum of *CaCO_3_* and *Ca(OH)_2_* in the reference samples is less for samples dried with acetone compared to isopropanol, which indicates that free water as well as parts of the solvent remain in the samples dried with acetone. This effect may cause problems in the interpretation of TGA data.

**Figure 4:**
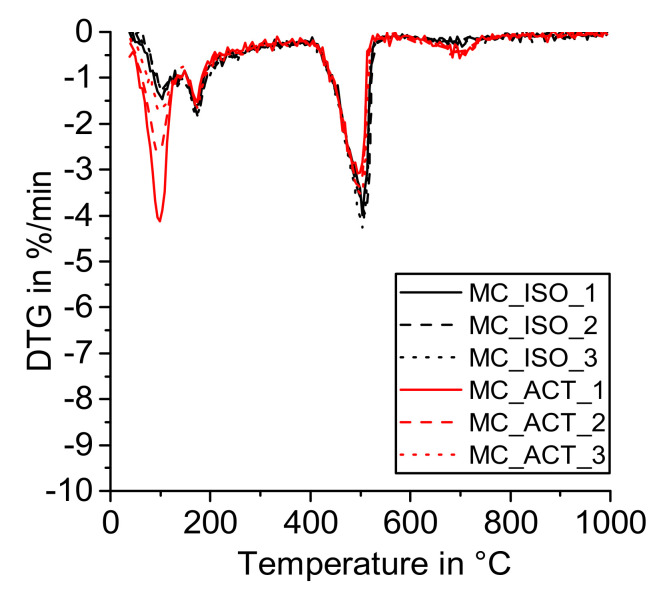
DTG—R^3^ sample with mixed calcined clay (MC) dried with isopropanol (ISO) and acetone (ACT).

**Figure 5:**
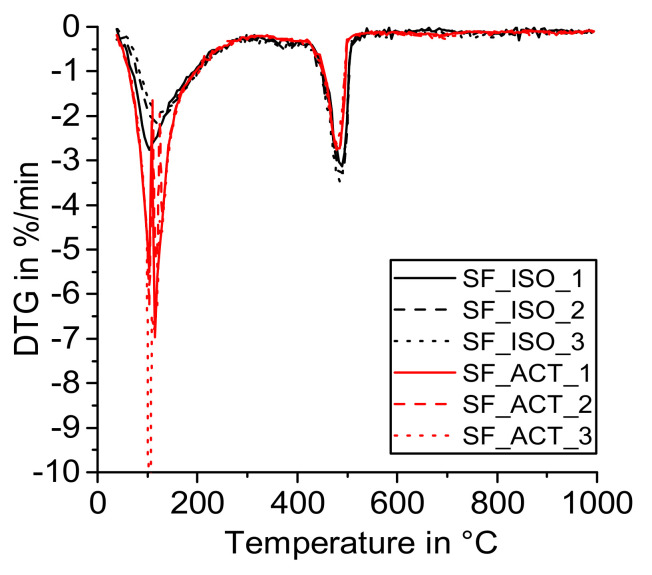
DTG—R^3^ sample with silica fume (SF) dried with isopropanol (ISO) and acetone (ACT).

**Figure 6:**
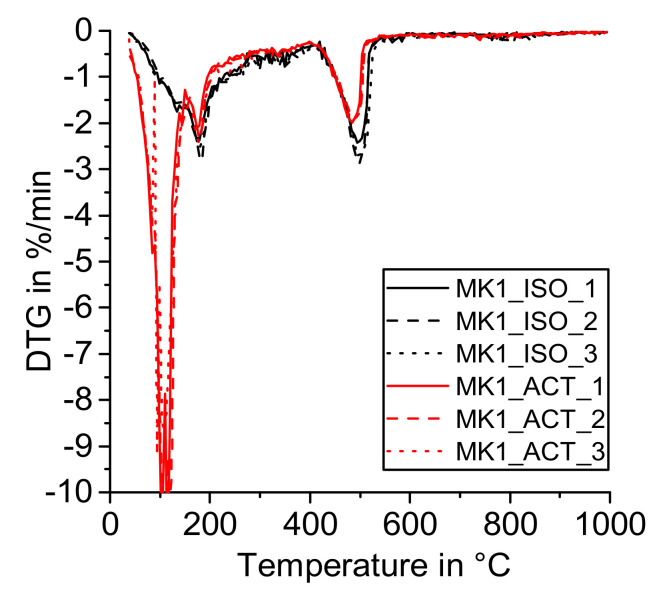
DTG—R^3^ sample with metakaolin (MK1) dried with isopropanol (ISO) and acetone (ACT).

**Figure 7:**
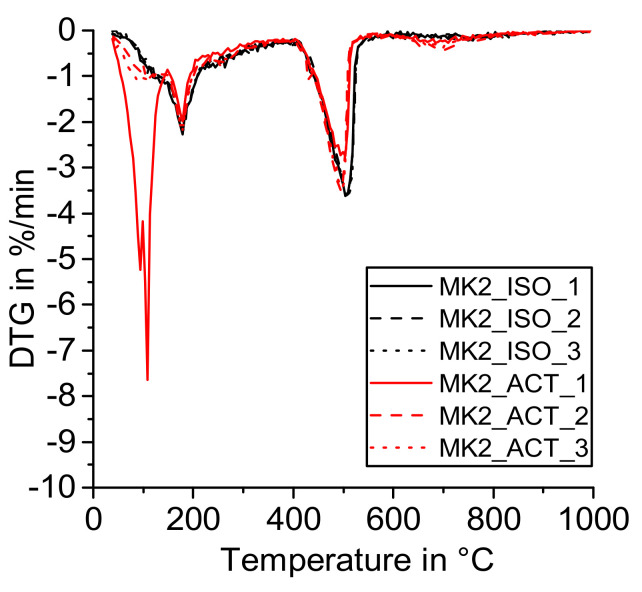
DTG—R^3^ sample with metakaolin (MK2) dried with isopropanol (ISO) and acetone (ACT).

Figure 4, Figure 5, Figure 6 and Figure 7 show the DTG curves of the R^3^ samples with silica fume (SF), metakaolin (MK1 and MK2), and mixed calcined clay (MC). Especially, the results with mixed calcined clay (Figure 4) support the observation investigated in the reference sample that drying with acetone increases the formation of carbonates (mass loss from 600 to 750 °C). Similarly, Kim and Olek showed enhanced carbonation in acetone-treated cement paste samples [24]. For stronger pozzolanic reactions like for silica fume and metakaolin, more Ca(OH)_2_ is consumed by SCM and consequently less Ca(OH)_2_ remains for carbonation. As the investigated mixed calcined clay (MC) is the weakest pozzolan in this study, the effect of the remaining free water when dried with acetone is visible for these samples similarly to the reference one. When looking at the mass loss from 40 to 105 °C, Figure 4 clearly shows that free water remains in the samples after drying with acetone. It decreases with time in the exicator as the mass loss reduces with the sequence CC_ACT_1, CC_ACT_2, and CC_ACT_3.

Some samples that were dried with acetone show strong deviations in the DTG curve in the temperature range of around 40 to 150 °C (e.g., Figure 5). This could be due to some remaining acetone or adol polycondensated products [35] in the samples that may affect the thermogravimetric measurement. Overall, the measured TGA data show a larger mass loss from room temperature to 40 °C for samples dried with acetone compared to isopropanol, which supports the results outlined above that some acetone (derived products [35]) and free water remained in these samples before testing.

**Figure 8:**
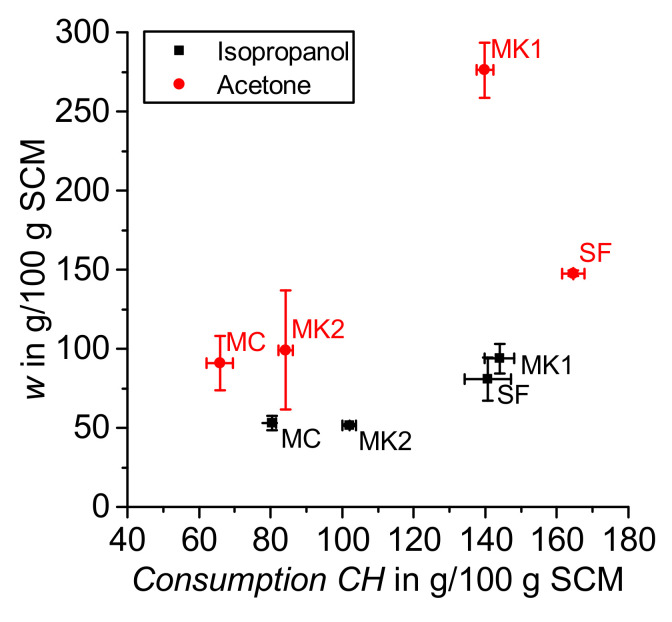
*w* and *Consumption CH* (acetone and isopropanol).

The results achieved from evaluation of the TGA data following the above explained mass balance approach are shown in Figure 8 for both drying with acetone and isopropanol. The value *w*, which summarizes the amount of physically and chemically bounded water (without water in calcium hydroxide) as well as the remaining organic solvents and free water, is larger in samples dried with acetone compared to isopropanol. In the literature, this value is often used to describe the amount of ‘chemically’ bounded water in the reaction products of cement paste samples [12,15,23], but as outlined above, R^3^ samples dried, e.g., with acetone or isopropanol (according to the described procedure) still contain some remaining organic solvent and free water that is per definition included in the calculation of the value *w* according to Equation (6). Infrared spectroscopy data from Zhang and Scherer [37] demonstrate that isopropanol also remains strongly sorbed by cement hydration products after oven drying. The comparison of the drying methods is not strictly focused on the effect of isopropanol vs. acetone, as the drying procedures are done differently. The purpose of the comparison in this paper is on the effect of the methods. This study considers the effect of drying R^3^ samples (with isopropanol and acetone done in different ways) on the results achieved with thermogravimetric analysis (TGA) using a newly proposed mass balance calculation approach.

Following the results of *Consumption CH* and drying with acetone (red symbols in Figure 8), pozzolanic reactivity could be rated as SF > MK1 > MK2 > CC. Even though the proposed mass balance approach considers carbonation, the results differ for the two solvents employed. This finding is in contrast with the observations of Kim and Olek for cement paste samples [24]. They reported nearly identical results for the amount of Ca(OH)_2_ when using the modified interpretation of TGA data analogous to the proposed mass balance approach in this study [24]. Moreover, Kim and Olek also stated that if significant carbonation takes place, it cannot be compensated by a modified interpretation of the thermogravimetric data [24]. These findings suggest that an appropriate choice of drying procedure is essential to achieve reliable thermogravimetric results in cement pastes as well as in R^3^ samples.

**Figure 9:**
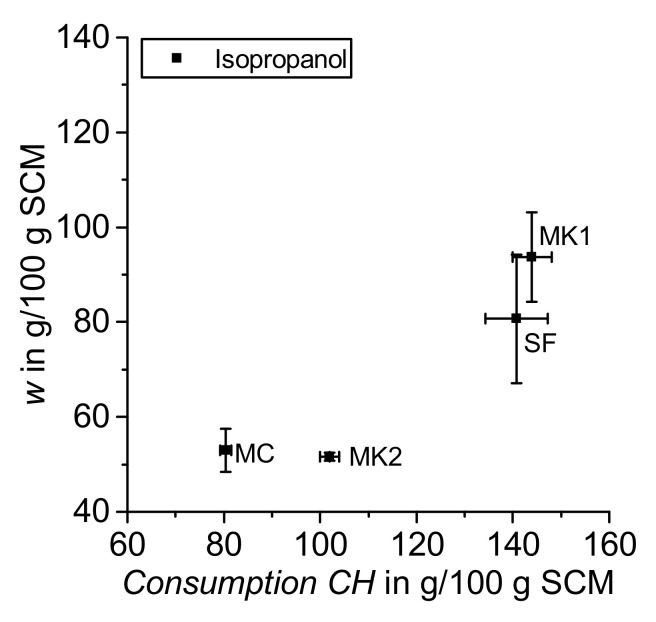
Zoom in of Figure 8: *w* and *Consumption CH* (isopropanol).

Figure 9 shows a zoom in of Figure 8 for the samples dried with isopropanol. It can be noted that the distribution of values is smaller when evaluating *Consumption CH* compared to *w* referring to the standard deviation shown in Figure 9. This finding is in line with the results for cement paste samples from Snellings et al. [12]. In a round robin test, they found that interlaboratory reproducibility was better for the analysis of calcium hydroxide content compared to the amount of chemically bounded water determined by thermogravimetric analysis [12], which corresponds with the calculation of the value *w* in this study for R^3^ samples. Snellings et al. explained this phenomenon by the larger sensitivity of the bounded water content to changes in the drying procedure of the samples [12]. Additionally, the value *w* calculated according to Equation (6) also includes the amount of remaining organic solvents and free water in the sample, which is strongly influenced by the drying procedure. Regarding *Consumption CH* and drying with isopropanol (black symbols in Figure 8 and Figure 9), pozzolanic reactivity can be ordered as MK1 > SF > MK2 > CC. 

**Figure 10:**
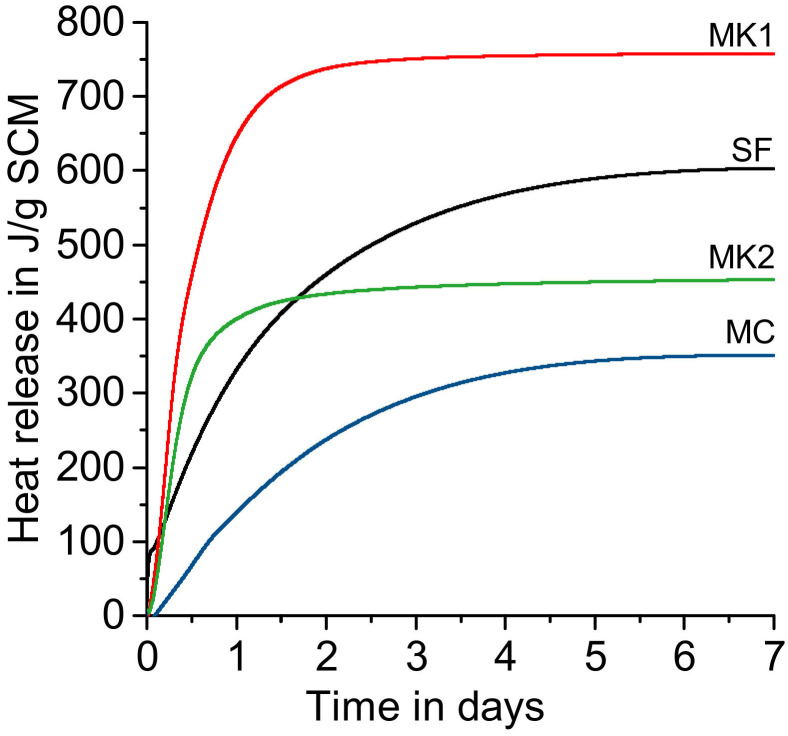
Results of the calorimetry test of R^3^ samples.

This sequence could be validated with calorimetry results as shown in Figure 10. The total heat release up to seven days at 40 °C for all tested SCMs is in the range of 350 J/g SCM for the mixed calcined clay (MC) and 757 J/g SCM for the relatively pure metakaolin employed in this study (MK1). The tested silica fume has a total heat release of 603 J/g SCM (SF) and the metakaolin with quartz impurities 452 J/g SCM (MK2). Consequently, all of the tested SCMs can be categorized in a high range of heat release (> 250 J/g SCM) according to Snellings and Kamyab [11]. Compared to the other pozzolans in this study, the mixed calcined clay (MC) shows the weakest reactivity. During calcination of clays, kaolinite is transformed to amorphous material, with a 2:1 ratio of the clays staying semi-crystalline. It is known that the clay structure significantly affects their pozzolanic reactivity [21]. More specifically, calcined 1:1 (sandwich layer = tetrahedral-octahedral = TO) clays like metakaolin are more reactive than calcination of other clays, such as illite, muscovite (mica) (2:1, TOT), and chlorite (2:1:1, TOTO) [21,38,39,40]. 

The calorimetric results in Figure 10 clearly show an early reaction of the two tested metakaolin (MK1 and MK2) compared to the heat development of the mixed calcined clay (MC) and silica fume (SF). The enhanced initial pozzolanic reaction of metakaolin that occurs within the first 48 h was already reported by Beuntner [21] by comparing calorimetric measurements of a relatively pure metakaolin and a mixed calcined clay. Higher reactivity could be attributed to the more dissolvable Al from metakaolin. The Si/Al ratio in solution may also play a decisive role in the reaction kinetics, in addition to the total content of the reactive phase. More reactive aluminate species may lead to an increase in reactivity [41]. This is also known in the process of the polycondensation reaction, in which aluminate species react more rapidly with silicate species than the reaction between two silicate species [42].

**Figure 11:**
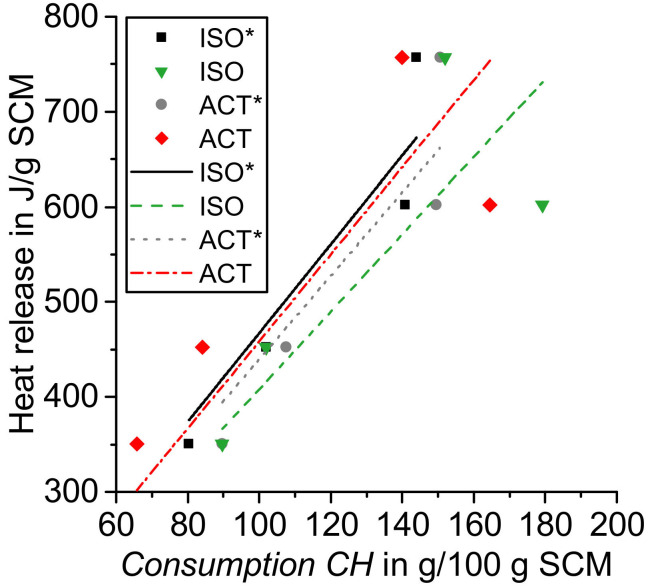
Correlation of heat release and *Consumption CH* (acetone and isopropanol) — Used abbreviations are outlined in Table 3.

Figure 11 shows the correlation of *Consumption CH* with the overall heat release from the calorimetry test samples dried with acetone and isopropanol and evaluated with the proposed mass balance approach (*) as well as without (*Consumption CH (Common)*) calculation of the calcium hydroxide consumption without considering the carbonation and TGA data of the original SCMs.

**Figure 12:**
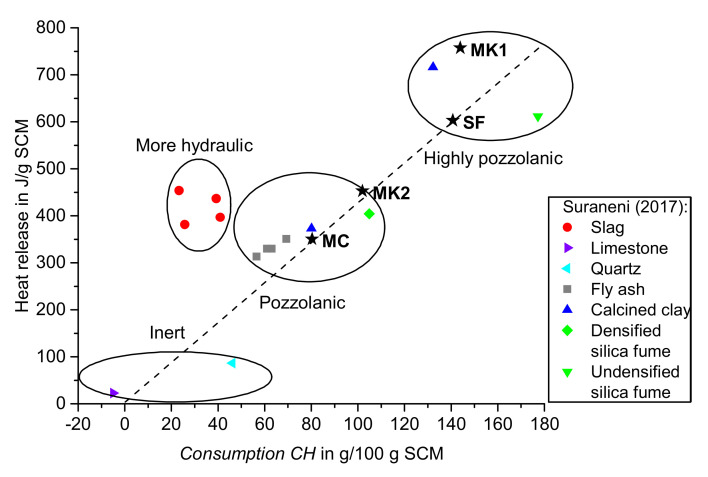
Results of this study SF, MK1, MK2, and MC (ISO*: Dried with isopropanol and calculated according to the proposed mass balance approach) shown in the figure adapted from Suraneni and Weiss [9].

Table 3 summarizes the regression accuracy values R^2^ from the linear correlation of *Consumption CH* and total heat release. The intersection with the y-axis was set to zero, similar to the approach from Suraneni and Weiss [9]. Drying with isopropanol results in a slightly higher R^2^ value compared to the reaction stoppage with acetone. When the mass balance approach of TGA data is applied, R^2^ is even higher as compared to the commonly used technique without taking into account the carbonation and TGA data of the original SCM. When comparing these two evaluation techniques, the percentual increase of R^2^ is a little higher for acetone-treated samples (3.2 %) compared to isopropanol (2.8 %). As drying with acetone results in increased carbonation, incorporating these effects in the evaluation of TGA data results in a somehow larger effect as for drying with isopropanol with a lesser amount of CaCO_3_. Data from Suraneni and Weiss show an R^2^ value of 0.94 for the linear regression of the same parameters generated with tests on different SCMs, as summarized in Figure 12 [9]. The amount of carbonates and TGA data of the original SCM in the interpretation of their thermogravimetric test results was not explicitly taken into account [9].

Table 3 shows also the calculated gradients for the different linear fits, and show a range between 4.08 (ISO) and 4.67 (ISO*). Taking into account the drying method with isopropanol and the proposed mass balance approach of this study (ISO*), the results indicate a heat of reaction of 467 J/g for the reacted calcium hydroxide, which refers to 34.56 kJ/mol calcium hydroxide for the tested SCMs. This result is in very good (8% relative error) agreement with Suraneni and Weiss’ calculated value of 31.67 kJ/mol based on their test results [9]. Newman reported a heat of reaction for the reaction of calcium hydroxide with silica gel and water of 10.3 kcal, which corresponds to around 43 kJ/mol [43]. The value for the reaction enthalpies between SiO_2_ and/or Al_2_O_3_ and calcium hydroxide during a pozzolanic reaction of SCMs is mainly dependent on the exact reaction that takes place. Reaction enthalpies calculated with the thermodynamic software GEMS are in the range of 26 to 35 kJ/mol [9]. 

Figure 12 shows the test results of this study, dried with isopropanol and calculated with the new proposed mass balance approach for TGA data (ISO*), along with the results from Suraneni and Weiss [9]. The SCMs analyzed in this study (marked with stars and labelled in bold in Figure 12) are mainly located directly on the linear fitted line through the data from these authors [9]. Following the classification proposed by Suraneni and Weiss, the calcined clay (MC) and the metakaolin with quartz impurities (MK2) are classified as pozzolanic SCMs [9]. The investigated silica fume (SF) as well as the relatively pure metakaolin (MK1) are located in the upper pozzolanic area. MK1, which has the highest amount of Al_2_O_3_ (42.18 wt.%) of the investigated SCMs, is located above the dashed line, which confirms the outcome from Suraneni and Weiss [9]. They concluded that the pozzolanic reaction of aluminum-rich phases releases more heat compared to silicon-rich ones [9]. 

## 4. Conclusions

This study considered the influence of drying R^3^ samples with isopropanol and acetone (done in different ways) on the results achieved with thermogravimetric analysis (TGA) using a new calculation approach. A mass balance approach was proposed for the measured thermogravimetric (TG) data from R^3^ tests that considers both the carbonation of samples and the TG data of the original SCMs. From the results of this study, the following conclusions can be drawn:

1. The drying procedure with acetone described in this paper resulted in some free water, sorbed acetone, and/or acetone derived organic polycondensates [35] remaining in the samples, which may affect the TGA data and its interpretation.
2. The TG results of the dried samples with the isopropanol method showed less carbonates and a better correlation with the calorimetric test results compared to samples dried with the acetone method. Thus, the drying method with isopropanol (instead of acetone) and use of the proposed mass balance approach is recommended for TGA of R^3^ tests.
3. The order of reactivity is the same for calorimetric measurement and TGA regarding the consumption of calcium hydroxide in samples dried with isopropanol: MK1 > SF > MK2 > CC.
4. The incorporation of carbonation and TGA data of original SCMs in the evaluation of TG data of R^3^ samples improves the correlation between calcium hydroxide consumption from TGA and the heat release determined with calorimetric measurement.

## Figures and Tables

**Table 1:** Chemical composition of SCMs in wt.% (evaluated on pressed tablets by X-ray fluorescence analysis).

SCM	SiO_2_	Al_2_O_3_	Fe_2_O_3_	CaO	MgO	Na_2_O	K_2_O	TiO_2_	Other
SF	97.02	0.55	0.21	0.32	0.49	0.18	0.98	0.00	0.25
MK1	52.95	42.18	2.38	0.05	0.07	0.00	0.31	1.77	0.29
MK2	58.80	32.79	3.50	2.35	0.17	0.06	0.35	1.80	0.18
MC	51.44	22.31	9.95	7.01	2.78	0.34	3.49	1.19	1.49

**Table 2:** Composition of 100 g of alkaline R^3^ emulsion.

Ca(OH)_2_	K_2_SO_4_	KOH	H_2_O
37.77 g	1.48 g	0.32 g	60.43 g

**Table 3:** Summary of the linear correlation of heat release = x · *Consumption CH*.

Drying	Mass Balance Approach of TGA Data	Abbreviation	R^2^	Gradient x
Isopropanol	New proposal	ISO*	0.988	4.67
Isopropanol	Common	ISO	0.961	4.08
Acetone	New proposal	ACT*	0.985	4.40
Acetone	Common	ACT	0.954	4.58

## Data Availability

The data presented in this study are available on request from the corresponding author.
